# Establishment of a Multilocus Sequence Typing Scheme for the Characterization of *Avibacterium paragallinarum*

**DOI:** 10.3390/vetsci11050208

**Published:** 2024-05-09

**Authors:** Mengjiao Guo, Yikun Jin, Haonan Wang, Xiaorong Zhang, Yantao Wu

**Affiliations:** 1 Jiangsu Co-Innovation Center for Prevention of Animal Infectious Diseases and Zoonoses, College of Veterinary Medicine, Yangzhou University, Yangzhou 225009, China; guomj@yzu.edu.cn (M.G.); mz120201498@stu.yzu.edu.cn (Y.J.); mx120220985@stu.yzu.edu.cn (H.W.); zxr@yzu.edu.cn (X.Z.); 2 Joint International Research Laboratory of Agriculture & Agri-Product Safety, Yangzhou University (JIRLAAPS), Yangzhou 225009, China

**Keywords:** *Avibacterium paragallinarum*, multilocus sequence typing, housekeeping gene, polymorphism

## Abstract

**Simple Summary:**

Infectious coryza is an acute respiratory infectious disease of chickens caused by *Av. paragallinarum*. *Av. paragallinarum* can be serotyped into A (A1–A4), B (B-1), and C (C1–C4) serovars. However, there is no effective molecular typing scheme to gain basic knowledge about the *Av. paragallinarum* population structure. MLST has played a major role in investigating the genetic structure of bacterial populations and has rapidly become the cornerstone technique for the molecular typing of pathogenic microorganisms. In this study, we are the first to establish this method and applied to PubMLST for a public MLST site for *Av. paragallinarum*. More samples and the ST sequence data can help to clearly observe the genetic evolution and epidemic patterns of *Av. paragallinarum*.

**Abstract:**

Infectious coryza is an acute respiratory infection caused by *Avibacterium paragallinarum*, which is widely distributed throughout the world. However, there is no effective molecular typing scheme to obtain basic knowledge about the *Av. paragallinarum* population structure. This study aimed to develop a multilocus sequence typing (MLST) scheme for *Av. paragallinarum* that allows for the worldwide comparison of sequence data. For this purpose, the genetic variability of 59 *Av. paragallinarum* strains from different geographical origins and serovars was analyzed to identify correlations. The MLST scheme was developed using seven conserved housekeeping genes, which identified eight STs that clustered all of the strains into three evolutionary branches. The analytical evaluation of the clone group relationship between the STs revealed two clone complexes (CC1 and CC2) and three singletons (ST2, ST5, and ST6). Most of the isolates from China belonged to ST1 and ST3 in CC1. ST8 from Peru and ST7 from North America together formed CC2. Our results showed that the *Av. paragallinarum* strains isolated from China had a distant genetic relationship with CC2, indicating strong regional specificity. The MLST scheme established in this study can monitor the dynamics and genetic differences of *Av. paragallinarum* transmission.

## 1. Introduction

*Avibacterium paragallinarum*, a member of the Pasteurellaceae family, is a Gram-negative bacterium responsible for infectious coryza (IC) in chickens, causing significant economic losses to the poultry industry [1]. The clinical course of IC typically includes severe respiratory tract infection with nasal discharge, facial swelling, sneezing, and infraorbital sinus swelling, resulting in growth retardation and reduced egg production [1]. In general, the symptoms persist for two to three weeks [2]. Asymptomatic infected birds and carriers play a crucial role in the spread of the disease [2,3]. Chickens serve as natural hosts for *Av. paragallinarum*. There are two typing schemes for *Av. paragallinarum*: the Page scheme and the Kume scheme. The Page scheme divides *Av. paragallinarum* into three serovars, A, B, and C, based on the plate agglutination assay [4]. Kume classified *Av. paragallinarum* into three distinct groups based on the hemagglutination inhibition (HI) test [5]. Blackall et al. regrouped and renamed the Kume protocol to match the Page serovars of A, B, and C, and subdivided it into nine serovars, A-1 to A-4, B-1, and C-1 to C-4 [6]. The Page scheme remains the most commonly used serotyping scheme for *Av. paragallinarum*, as inactivated vaccines only protect against the Page serogroup present in the vaccine [7,8,9]. The Page scheme requires three serovar-specific antibodies, whereas the Kume scheme requires nine serovar-specific antibodies, all of which are complicated and require chicken erythrocytes fixed with glutaraldehyde and antigens treated with potassium thiocyanate or hyaluronidase [10]. Consequently, only a handful of laboratories that specialize in relevant research can achieve the serovar classification of *Av. paragallinarum* [11]. In addition, some isolates fail to display HA activity even after undergoing hyaluronidase treatment and, therefore, cannot be serotyped by the HI test [12].

IC occurs globally, including in China, Japan, South Korea, India, Bangladesh, and Thailand in Asia; the United Kingdom and the Netherlands in Europe; South Africa in Africa; Mexico and the United States in North America; and Peru in South America. The serovars A, B, and C of *Av. paragallinarum* strains are widely distributed, and the epidemic strains differ between countries. In China, serovar A was first reported in 1987, serovar C in 1993, and serovar B in 2003 [13]. Before 2000, most of the isolated strains were of serovar A. Since 2000, serovar C strains were isolated gradually. Between 2012 and 2017, serovar B was the most common type isolated in China [13,14]. In recent years, serovars A and C have become more prevalent. The incidence of IC has risen in China, and the disease has even occurred in chickens immunized with inactivated vaccines, indicating that the inactivated vaccine has insufficient immune protection against the current epidemic strains [15]. A previous study showed that the variant strains and increased virulence lead to decreased inactivated vaccine protection [15]. It is difficult to compare strains around the world and exchange information across institutes. To effectively control the spread of *Av. paragallinarum*, it is essential to implement rapid, accurate, and convenient strain typing methods to accurately determine its evolutionary trajectory.

Sakamoto et al. proposed a multiplex PCR and PCR-RFLP method based on the hypervariable region of the HMTp210 gene for the serotyping of *Av. paragallinarum* [16]. However, it has been demonstrated that these methods are not suitable for differentiating the serovar of *Av. paragallinarum* isolates [11]. Soriano et al. claimed that ERIC-PCR could be used as a laboratory molecular typing tool for *Av. paragallinarum* [17]. Subsequently, this method was also evaluated, and the data indicated that ERIC-PCR is not ideal for the molecular typing of *Av. paragallinarum* serovars [18]. Multilocus sequence analysis (MLSA) of *Avibacterium* was conducted to classify *Pasteurellaceae*. The authors highlight the advantages of MLSA over 16S rRNA sequence analysis. It was found that *Av. paragallinarum* is a distinct species, while *Av. volantium*, *Av. avium*, *Av. gallinarum*, *Av. endocarditis*, and *Avibacterium* sp. A form a complex of species that are difficult to distinguish. MLSA is suitable for the initial typing and phylogenetic analysis of new populations [19]. Another scheme, multilocus sequence typing (MLST), is suitable for the fine typing of known populations. MLST is an ideal choice for long-term epidemiological monitoring and global strain comparison due to its standardization and ease of data sharing. There is currently no mature molecular typing method for *Av. paragallinarum*.

MLST is a method that utilizes the well-established principles of multilocus enzyme electrophoresis [20], which detects amino acid sequence changes in enzymes associated with the activity of basic cell families [21]. MLST determines the genetic information of each strain through the internal fragments of seven housekeeping genes, each about 500 bp nucleotides in length. The different sequences of each gene are referred to as alleles, and the combination of seven alleles creates an allelic map that defines the sequence type (ST). The housekeeping genes are highly conserved, making it a slow process for mutations to occur. As a result, MLST offers a reliable and precise method for evaluating isolates with high resolution. This technique has proven to be an invaluable tool for analyzing genetic relationships between strains and can be applied to study the phylogeny and molecular epidemiology of pathogens [22].

MLST allows researchers to compare data from different laboratories around the world for the same pathogenic microorganism quickly and efficiently [23]. This method has played a major role in investigating the genetic structure of bacterial populations and rapidly became the cornerstone technique for molecular typing of pathogenic microorganisms [24]. Since the establishment and application of MLST in *Neisseria meningitidis* in 1998 [25], MLST has been applied to a wide range of bacteria and has become the gold standard for bacterial molecular typing [26]. However, it has not yet been implemented in *Av. paragallinarum*. In this study, we developed an MLST typing method for *Av. paragallinarum* based on strains isolated in recent years and whole-genome data from the GenBank. We applied to PubMLST for a public MLST site of *Av. paragallinarum* for reference and use by other researchers. 

## 2. Materials and Methods

### 2.1. Bacterial Isolates and Strains

A total of 59 strains of *Av. paragallinarum* were used to develop the MLST, including 44 field strains, 2 reference strains, and 13 complete genome sequences (Table 1). *Av. paragallinarum* strains (29 strains) were isolated from the chickens with clinical symptoms of IC and conserved in our laboratory [27]; 14 strains were isolated from the infraorbital sinuses of chickens with clinical symptoms of IC in this study. Additionally, strain 2021/06 was sent to us by a biological company in June 2021 for serotyping. Most of the field strains were isolated from Jiangsu, while a few were isolated from Ningxia, Hebei, and Hubei. Reference strains 221 and H-18 were purchased from the China Veterinary Culture Collection Center (CVCC). The complete genome sequences of 13 strains were obtained from GenBank. The details of all 59 *Av. paragallinarum* strains are shown in Table 1.

*Av. paragallinarum* isolates were cultured on trypticase soy agar (Hopebio Biotech Co., Ltd., Qingdao, China) with 5% fetal bovine serum (Lonsera Biotech Co., Ltd., Suzhou, China) and 0.0025% reduced nicotinamide adenine dinucleotide (Sangon Biotech Co., Ltd., Shanghai, China) at 37 °C in 5% CO_2_ for 24–48 h. The colony morphology was translucent with a diameter of approximately 2 mm. The specific Hpg-2 primers (F: TGAGGGTAGTCTTGCACGCGAAT; R: CAAGGTATCGATCGTCTCTCTACT) were used to identify *Av. paragallinarum* [28]. The serovar of the isolates was determined by the HI test [12]. The antisera for the HI test were obtained from our previous study [27]. The isolates’ culture was centrifuged and treated with potassium thiocyanate (Sinopharm Chemical Reagent Co., Ltd., Shanghai, China) at 4 °C for 2 h. Then, the isolates were sonicated and washed with PBS as antigens. The chicken erythrocytes were fixed with glutaraldehyde (Sinopharm Chemical Reagent Co., Ltd., Shanghai, China). The serovar of the isolate corresponds to the antiserum with the highest HI titer. 

### 2.2. Selection of Housekeeping Genes for MLST

Based on the MLST analysis of Pasteurellaceae obtained from the PubMLST database (https://pubmlst.org/organisms, accessed on 27 September 2022) [29], 14 housekeeping genes were selected from the genomic sequence of *Av. paragallinarum*. The primers were designed to target conserved regions within these sequences, and partial segments within the genes were delineated using Primer5 software (Version 5.0). Based on the determination principles of housekeeping genes, 7 genes were ultimately identified by comparing the genomic sequence of *Av. paragallinarum*. The primers are listed in Table 2. The positional distribution of the housekeeping genes on the chromosome was as follows: *pmi*, 248.0 kb; *infB*, 632.4 kb; *mdh*, 708.1 kb; *adk*, 1316.9 kb; *deoD*, 1792.6 kb; *recA*, 2432.3 kb; *zwf*, 2489.2 kb. The minimum distance between the housekeeping genes was 56.9 kb.

### 2.3. Gene Amplification and Sequencing

PCR was performed in a total volume of 25 μL containing 2.5 μL of 10× *EasyTaq*^®^ buffer, 2 μL of 2.5 mM dNTPs, 0.5 μL of forward and reverse primers (10 μM), 0.25μL of *EasyTaq*^®^ DNA Polymerase (Transgen Biotech Co., Ltd., Beijing, China), and 1 μL of genomic DNA templates. The thermocycling parameters were as follows: initial denaturation at 94 °C for 5 min, followed by 94 °C for 30 s, 55 °C for 30 s, and 72 °C for 60 s for 35 cycles, completed with a final extension at 72 °C for 7 min. The PCR products were sent to General Biol Co., Ltd. (Anhui, China) for sequencing. The obtained sequences were edited for further analysis.

### 2.4. MLST Analysis

For each housekeeping gene, the nucleotide sequences of 59 strains were compared, and the different sequences were assigned allele numbers (different alleles were numbered in ascending order). The *Av. paragallinarum* strains were characterized by distinct allelic combinations of 7 loci, which defined the allelic profile. The distinct allelic profiles were designated STs. The number of polymorphic sites and the number of alleles were calculated using DnaSP software (Version 5.10.0001). The average GC content was calculated using DNASTAR software (Version 7.1.0.44). Pairwise ratios of nonsynonymous substitutions to synonymous substitutions (*dN*/*dS*) were calculated by the method of modified Nei–Gojobori with the calculated R of the MEGA X software (Version 10.2.6) [30]. The neutral theory of molecular evolution test for housekeeping genes was performed using the statistical method Tajima’s D in DnaSP [31,32].

### 2.5. Phylogenetic Analysis of STs

The internal fragments of the 7 housekeeping genes were concatenated using Snapgene (Version 4.2.4). Phylogenetic analysis of the concatenated sequences was performed by the neighbor-joining (NJ) method using MEGA X software with 1000 bootstrap replications to assess support for the various groups. The clonal complexes were identified by the goe BURST (Version 1.2.1) program according to the STs [33]. Split decomposition trees were generated by using splits tree (Version 4.18.1).

## 3. Results

### 3.1. MLST Analysis

The *pmi*, *infB*, *mdh*, *adk*, *deoD*, *recA*, and *zwf* genes were identified as the housekeeping genes of the *Av. paragallinarum* MLST scheme. All of these genes are present in a single copy in *Av. paragallinarum* genomes, and the length range of the fragments used for typing was 432–864 bp (Table 2). The average G+C content of the housekeeping gene fragments ranged from 44 to 48%. The number of polymorphic sites varied significantly among the seven housekeeping genes. The partial sequences of the genes *adk* and *pmi* revealed a minimum and maximum of 2 and 109 polymorphic sites, respectively. For 59 *Av. paragallinarum* strains, the number of alleles for the housekeeping genes was as follows: three (*pmi* and *adk*), four (*mdh* and *deoD*), five (*recA* and *zwf*), and six (*infB*). The nucleotide polymorphism is the average number of nucleotide differences between any two alleles in a population and is used to measure the degree of difference in genetic information in a population. The nucleotide polymorphisms of the seven housekeeping genes ranged from 0.00015 (*adk*) to 0.02823 (*pmi*). The *dN*/*dS* values ranged from 0 to 0.80, all less than 1. The Simpson’s index of diversity of the housekeeping genes ranged from 0.067 to 0.613, with *adk* having the lowest value of 0.067 and *zwf* having the highest value of 0.613. The neutral theory test results revealed that the D values of *pmi*, *adk*, and *recA* were all less than 0, while the D values of the remaining four genes were greater than 0. All the housekeeping genes did not deviate significantly from 0. These data are presented in Table 3. 

The seven housekeeping genes of the strain 2019JS28 were taken as allele 1 for each gene, forming ST1. According to the nucleotide sequence analysis of the seven housekeeping genes, 59 strains of *Av. paragallinarum* were classified into eight ST types. The highest proportion was ST1 (34 strains) with 57.63% of the total number of isolates, followed by ST3 (11 strains) with 18.64% and ST8 (9 strains) with 15.25%. ST2, ST4, ST5, ST6, and ST7 each had only one strain (Table 4). Based on the above results, we applied to PubMLST for a public MLST site for *Av. paragallinarum* (https://pubmlst.org/organisms/avibacterium-paragallinarum, accessed on 21 March 2024). 

### 3.2. Phylogeny and Cluster Analysis of STs

**Figure 1:**
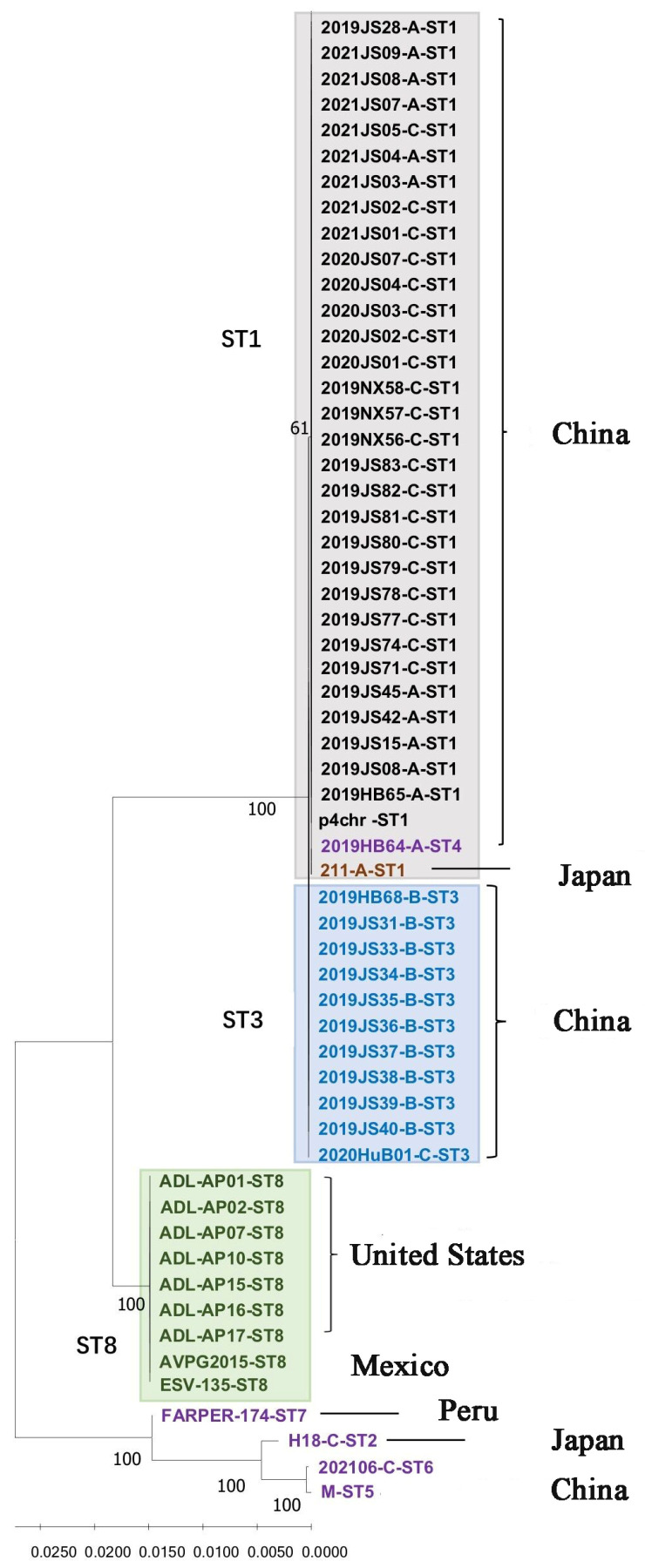
Phylogenetic tree showing the relatedness of 59 *Av. paragallinarum* strains generated from MLST sequences.

After connecting the seven housekeeping genes in the order of *pmi*, *infB*, *mdh*, *adk*, *deoD*, *recA*, and *zwf*, the phylogenetic tree was constructed. As shown in Figure 1, three major branches were observed for the 59 *Av. paragallinarum* strains. Most of the isolates from China belonged to one group, with the exception of the 2021/06 and M strains, which showed a marked genetic distance from the other strains from China. All isolates from North America, including Mexico and the United States, belonged to the same branch. The genetic distance between the last branch and the above two evolutionary branches was relatively far, and it was further divided into two small evolutionary branches. The FARAPER-174 strain belonged to a small branch, and the other branch included the 2021/06, M, and H-18 strains. The *Av. paragallinarum* strains used for MLST analysis in this study were isolated between 2008 and 2021, as well as two reference strains 221 and H-18 from earlier years. Nineteen strains isolated in 2019, five strains in 2020, nine strains in 2021, and the reference strain 221 were assigned to ST1. Seven strains from 2019, one strain from 2015, and one strain from 2008 were assigned to ST8. In addition, ST1 contained serovars A and C, and ST3 was mainly serovar B. The serovars of ST8, ST4, and ST5 were unknown.

**Figure 2:**
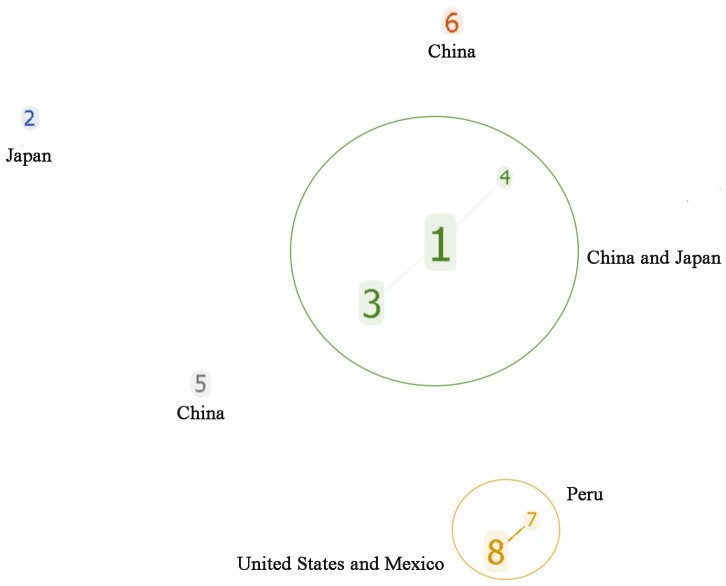
Cluster analysis for STs of 59 *Av. paragallinarum* strains.

The analytical evaluation of the clone group relationship between the STs showed that MLST formed two clone complexes (CC1 and CC2) and three singletons (ST2, ST5, and ST6) (Figure 2). CC1 was a dominant clone complex (47 strains) composed of three ST types: ST1, ST3, and ST4, with ST1 being the central ST type. CC2 was composed of ST7 and ST8. Among the 44 *Av. paragallinarum* strains isolated in China, 43 strains belonged to CC1, and two strains belonged to independent types ST5 and ST6, respectively. The strains of CC2 were all from abroad; ST8 from North America, and ST7 from Peru. This indicates that the genome sequences of the *Av. paragallinarum* isolated from China were consistent and had a distant genetic relationship with foreign CC2, indicating strong regional specificity.

## 4. Discussion

The purpose of this study was to characterize *Av. paragallinarum* strains under standardized conditions by developing an MLST scheme. This scheme can be easily carried out by other laboratories and can compare *Av. paragallinarum* sequences globally. To achieve this idea, seven housekeeping genes were identified, and 59 *Av. paragallinarum* strains were sequenced and compared.

IC is a common infectious disease in chickens, characterized by acute onset and high infectivity. With the increasing intensification of chicken farms, the high bird density increases the pressure on the prevention and treatment of respiratory diseases such as IC [2]. At the same time, with the implementation of the drug reduction policy and the increase in the supervision and punishment of drug residues, antibiotics are strictly restricted, particularly during the laying period, which may be another reason for the rising incidence rate of IC in China [15,27]. Due to the lack of cross protection between different serovars, it is necessary to classify and identify epidemic strains to guide vaccine selection [34]. As mentioned earlier, the serological typing method based on HI test requires the standard serum of different serovars and can only be carried out by laboratories conducting relevant research [35]. Therefore, based on traditional bacterial isolation, there is an urgent need to develop a molecular typing method for *Av. paragallinarum*.

In this study, seven housekeeping genes (*pmi*, *infB*, *mdh*, *adk*, *deoD*, *recA*, and *zwf*) were used to establish the MLST method for typing *Av. paragallinarum*. These seven genes are relatively conserved and stable and are located throughout the chromosome of *Av. paragallinarum* in a relatively dispersed manner, which can avoid the influence of genetic linkage and reflect the phylogenetic relationship between different strains. To further demonstrate the feasibility of the housekeeping genes, the number of allele types, the number of polymorphic sites, nucleotide polymorphism, *dN*/*dS*, Simpson’s index, and neutral theory were evaluated. *Zwf* had the highest number of alleles, while *adk* and *pmi* had the lowest number of alleles. Simpson’s index is used to describe the probability that the number of individuals obtained from two consecutive samplings in a community belong to the same species. It is used to estimate the species diversity of the population. The Simpson’s index ranges from 0 to 1, with higher values indicating greater population diversity [36]. The Simpson’s index of *zwf* was the highest with 0.613, indicating the diversity of *zwf*. The number of polymorphic sites refers to differences in gene sequences at specific loci on the allele, which are considered to be early mutations that can be stably inherited and are indicative of the significance of the genetic evolution of the strain [37]. The polymorphism mutation rate reflects the degree of variation within the housekeeping genes, with the lowest mutation rate for *adk* ensuring the stability of MLST, and the highest for *pmi* ensuring the resolution of the MLST. The *dN*/*dS* values can determine the degree of selection pressure of the housekeeping genes [38]. The nucleotide polymorphism and *dN*/*dS* values indicate the stability of the housekeeping genes at the nucleotide and amino acid levels, respectively. In this study, the nucleotide polymorphism of the seven housekeeping genes was small (0.00015–0.02823), and the *dN*/*dS* values were all less than 1, indicating that the selected housekeeping genes met the requirements for conservation and stability. The neutral evolution theory suggests that biological evolution is mainly due to random genetic drift of neutral mutations in natural populations, independent of natural selection. The neutral theory test results showed that the values were close to 1, which proves that the selected housekeeping genes conformed to the neutral theory and were under little pressure from natural selection [32]. These findings indicate that the selected housekeeping genes can maintain their conservation and reflect the diversity of genetic site differences. The seven housekeeping genes had sufficient stability and resolution, which could meet the requirements for establishing the MLST method for *Av. paragallinarum*. 

The MLST analysis included strains from three continents and five different countries that were widely distributed across STs and phylogenetic clusters. The MLST based on seven housekeeping genes divided 59 strains of *Av. paragallinarum* into eight ST types, which were clustered into three evolutionary branches. Most of the isolates from China belonged to ST1 and ST3 in CC1. However, the strains 2021/06 and M belonged to ST6 and ST5 respectively, which were genetically distant from CC1. There could be several reasons for this, including genetic mutation, introduction from overseas, or native diversity. These isolates may have accumulated enough mutations over time. Secondly, globalization allows pathogens to spread rapidly between countries, so these strains could be the result of international transmission. To determine this result, we analyzed additional epidemiological data. Tracing the origin of these two strains showed that strain M was submitted to GenBank without any isolation information. Similarly, the original source of strain 2021/06 was also unknown. Since the original source information of these two strains is uncertain, based on the MLST results of this study, we speculate that strains 2021/06 and M may not have been isolated in China. All isolates from North America, including Mexico and the United States, were ST8 and belonged to the same branch. ST7 from Peru and ST8 from North America together formed CC2. *Av. paragallinarum* strains belonging to the same ST type are generally from the same geographical origin, indicating that a specific ST is associated with a geographical origin. ST1 contained the serovars A and C, and ST3 was predominantly the serovar B, suggesting a possible correlation between serovars and phylogenetic relationships. Through analysis, MLST can understand the genetic and evolutionary characteristics of *Av. paragallinarum* strains, providing useful information for the prevention and treatment of IC. However, the conclusions drawn from the available data are still very limited, which is directly related to the limitations of our sample size. We uploaded this MLST scheme to create a public database for *Av. paragallinarum*, collecting sequences and isolates from around the world. With more samples and ST sequence data, the genetic evolution and epidemic patterns of *Av. paragallinarum* can be clearly observed.

## 5. Conclusions

The MLST scheme established in this study can provide a clear, reproducible, and portable typing system in order to gain insight into the population structure of *Av. paragallinarum* by identifying genetic variation in bacterial sequences. Eight STs were identified by MLST and clustered into three evolutionary branches. In general, *Av. paragallinarum* strains from the same geographical region were separated by MLST and assigned to identical STs. We hope that more people can enrich and improve this MLST database and use the typing technology as a technical reserve for *Av. paragallinarum* monitoring and evolutionary analysis. In the future, this method can monitor the epidemic trend of *Av. paragallinarum* through genetic evolutionary traceability and compare the correlation between epidemics at different times in the same area or at the same time in different areas, so as to achieve more accurate prevention and control.

## Figures and Tables

**Table 1:** Background information of 59 *Av. paragallinarum* strains.

Strains	Country	Year	Serovar	Origin or GenBank Accession No.
221	Japan	/	A	CVCC
H-18	Japan	/	C	
2019/JS08, 2019/JS15, 2019/JS28, 2019/JS42, 2019/JS45, 2019/HB64, 2019/HB65	China	2019	A	[27]
2019/JS31, 2019/JS33, 2019/JS34, 2019/JS35, 2019/JS36, 2019/JS37, 2019/JS38, 2019/JS39, 2019/JS40, 2019/HB68	China	2019	B	
2019/NX56, 2019/NX57, 2019/NX58	China	2019	C	
2020/JS71, 2020/JS74, 2020/JS77, 2020/JS78, 2020/JS79, 2020/JS80, 2020/JS81, 2020/JS82, 2020/JS83	China	2020	C	
2020/HUB01, 2020/JS01, 2020/JS02, 2020/JS03, 2020/JS04, 2020/JS07	China	2020	C	This study
2021/JS01, 2021/JS02, 2021/JS05, 2021/06	China	2021	C	
2021/JS03, 2021/JS04, 2021/JS07, 2021/JS08, 2021/JS09,	China	2021	A	
FARPER-174	Peru	2015	C-2	CP034110.1
ESV-135	Mexico	2008	C-1	CP050316.1
ADL-AP01, ADL-AP02, ADL-AP07, ADL-AP10, ADL-AP15, ADL-AP16, ADL-AP17	United States	2019	unknown	CP051642.1, CP051641.1, CP051640.1, CP051639.1, CP051638.1, CP051637.1, CP051636.1
AVPG2015	Mexico	2014	unknown	CP058307.1
p4chr1	China	2021	unknown	CP081939.1
M	China	2010	unknown	CP086713.1
ZJ-C	China	2019	unknown	CP095161.1

**Table 2:** Primer sequences of 7 housekeeping genes.

Gene	Protein Product	Primer Sequence (5′–3′)	Fragment Size (bp) ^a^
*pmi*	Mannose-6-phosphate isomerase	Forward: TTACATTATCCGAACACGC	597 (863)
		Reverse: TTACCCATTAAACGGTCAGC	
*infB*	Translation initiation factor IF-2	Forward: TTTACCGTGGTCAACGTGTC	594 (831)
		Reverse: GAAAGAAAAGCGGCGGAAGA	
*mdh*	Malate dehydrogenase	Forward: CTAACTATTAATAAATTTCTCTCCTA	738 (936)
		Reverse: ATGAAAGTTGCTGTTTTAGGTGCTG	
*adk*	Adenylate kinase	Forward: ATGAAAATTATTCTTCTAGGTGCAC	447 (623)
		Reverse: TCGGCACTCACTGCTTCC	
*deoD*	Purine-nucleoside phosphorylase	Forward: GGGCTTTTGCTGATGTAGTATT	432 (717)
		Reverse: GGTTAGTTGGCGTTCTTCT	
*recA*	DNA recombination/repair protein	Forward: ATCACTACCCCAGAAGAAAAAGAA	864 (1063)
		Reverse: TATCCATTACATCATTATTGTCATT	
*zwf*	Glucose-6-phosphate dehydrogenase	Forward: CGTGCAATGAGTTTGTCCG	738 (1413)
		Reverse: TATCGTGATTTTTGGGGCAT	

^a^ The numbers in parentheses represent the gene’s size.

**Table 3:** Genetic diversity of housekeeping genes in 59 *Av. paragallinarum* strains.

Gene	Average G+C Content (%)	Number of Alleles	Number of Polymorphic Sites	Percentage of Variable Nucleotide Site (%)	Nucleotide Polymorphism	*dN*/*dS*	Simpson’s ID	Tajima’s D
*pmi*	44	3	109	18.26	0.02823	0.22	0.371	−1.08584
*infB*	45	6	18	3.08	0.00872	0.69	0.395	0.91678
*mdh*	48	4	8	1.09	0.00267	0	0.368	0.04274
*adk*	45	3	2	0.45	0.00015	-	0.067	−1.44562
*deoD*	45	4	31	7.17	0.01642	0.80	0.368	0.10658
*recA*	46	5	19	2.20	0.00365	0	0.369	−0.71009
*zwf*	46	5	35	4.76	0.01415	0.25	0.613	0.92331

**Table 4:** STs of 59 *Av. paragallinarum* strains.

Strain	ST	Allelic Profile						
		*pmi*	*infB*	*mdh*	*adk*	*deoD*	*recA*	*zwf*
221, 2019/JS08, 2019/JS15, 2019/JS28, 2019/JS42, 2019/JS45, 2019/NX56, 2019/NX57, 2019/NX58, 2019/HB65, 2019/JS71, 2019/JS74, 2019/JS77, 2019/JS78, 2019/JS79, 2019/JS80, 2019/JS81, 2019/JS82, 2019/JS83, 2020/JS01, 2020/JS02, 2020/JS03, 2020/JS04, 2020/JS07, 2021/JS01, 2021/JS02, 2021/JS03, 2021/JS04, 2021/JS05, 2021/JS07, 2021/JS08, 2021/JS09, p4chr1, ZJ-C	1	1	1	1	1	1	1	1
H-18	2	2	2	2	2	2	2	2
2019/JS31, 2019/JS33, 2019/JS34, 2019/JS35, 2019/JS36, 2019/JS37, 2019/JS38, 2019/JS39, 2019/JS40, 2019/HB68, 2020/HuB01	3	1	1	1	1	1	1	3
2019/HB64	4	1	1	1	1	1	1	4
M	5	2	2	3	3	3	5	7
2021/06	6	2	3	3	1	3	3	5
FARPER-174	7	2	4	4	1	4	4	6
ESV-135, ADL-AP01, ADL-AP02, ADL-AP07, ADL-AP10, ADL-AP15, ADL-AP16, ADL-AP17, AVPG2015	8	3	4	4	1	4	4	6

## Data Availability

The data presented in this study are available in this article.
